# The quality of stored umbilical cord and adult-donated whole blood in Mombasa, Kenya

**DOI:** 10.1111/j.1537-2995.2009.02489.x

**Published:** 2010-03

**Authors:** Oliver Hassall, Kathryn Maitland, Gregory Fegan, Johnstone Thitiri, Lewa Pole, Robert Mwakesi, Douglas Denje, Kongo Wambua, Kishor Mandaliya, Imelda Bates

**Affiliations:** Centre for Geographic Medicine Research (Coast), Kenya Medical Research Institute/Wellcome Trust Research ProgrammeKilifi, Kenya; the Department of Primary Health Care, University of OxfordOxford, UK; the Department of Paediatrics, Imperial College London, and the Department of Epidemiology and Population Health, London School of Hygiene and Tropical MedicineLondon, UK; the Coast Provincial General Hospital and the Regional Blood Transfusion CentreMombasa, Kenya; and the Liverpool School of Tropical MedicineLiverpool, UK

## Abstract

**BACKGROUND:**

In sub-Saharan Africa umbilical cord blood may be a useful source of blood for transfusion. Before clinical trials, evidence is needed that cord blood donations, which vary greatly in volume, can be collected and stored into a fixed volume of anticoagulant-preservative solution obviating the need for prestorage processing.

**STUDY DESIGN AND METHODS:**

Twenty-four umbilical cord whole blood (UC-WB) donations were collected into 21 mL of CPDA-1 and refrigerated for 35 days. The Kenya Blood Transfusion Service provided 12 adult-donated whole blood (AD-WB) controls. Supernatant hemoglobin (Hb) and potassium were assayed at 7-day intervals.

**RESULTS:**

UC-WB red blood cell hemolysis and potassium loss increased throughout storage but did not differ significantly with cord blood volume. Hemolysis rates did not differ significantly between UC-WB and AD-WB but UC-WB potassium loss was slightly but significantly greater than AD-WB on Days 2, 7, and 14 (p < 0.05). In the AD-WB controls, eight were low volume (<405 mL), two had total Hb of less than 45 g, and two showed hemolysis greater than 0.8% by Day 28.

**CONCLUSION:**

Variable volumes of UC-WB can be stored for 35 days without prestorage processing and further work into its suitability for transfusion to children is justified. The quality of conventional AD-WB is a concern and needs further evaluation.

In industrialized countries, stored autologous umbilical cord red blood cells (RBCs) have been infused back to neonates who require a transfusion because of surgery or anemia of prematurity. 1–3 Allogeneic cord blood transfusion in older children and adults has also been reported.^4^,^5^ In developing countries, where conventional blood supplies are severely limited but the demand for transfusions in young children is high, allogeneic cord blood may be a useful supplement to the blood supply if operational feasibility and safety can be demonstrated.^6^,^7^ We have previously published data relating to acceptability and consent^8^ and a large study to assess microbiologic safety including the risk of bacterial contamination is under way. Here we report on the quality of umbilical cord RBCs collected and stored as whole blood in a fixed volume of anticoagulant-preservative (AP) solution.

Cord blood is most simply collected by venipuncture of a suspended umbilical cord so that the blood flows by gravity into a collection bag prefilled with a fixed volume of AP solution. This is analogous to the manner in which a conventional adult blood donation is collected. Cord blood collection volumes rarely exceed 150 mL, so the collection bags are smaller than those for adult blood donations of 450 mL. The fixed volume of AP solution with which the collection bag is prefilled is also reduced, so that the blood-to-AP ratio is optimal for 150 mL of cord blood and no greater (21 mL of AP solution; 7:1 ratio). However, cord blood collection volumes are variable and are usually much less than 150 mL (mean, 64-86 mL^2^–^4^,^7^,^9^) and therefore the cord blood–to-AP ratio is also variable and usually higher than is considered optimal.

In industrialized countries, when cord blood has been transfused, this issue has been negated by prestorage processing of whole cord blood into RBCs by differential centrifugation, component separation into satellite bags, and the addition of an additive solution in a fixed ratio to the concentrated RBC fraction.^10^ However, the bag configuration, laboratory equipment, and expertise required to do this are technically and financially prohibitive for developing countries where most blood is stored and transfused as whole blood.^11^ In these countries the transfusion of cord blood is only likely to be feasible if it can be stored as whole blood without prestorage processing.

The purpose of this study therefore was to investigate the storage characteristics of whole cord blood collected into a fixed volume of AP solution under local conditions. We were unable to find published data on the storage of conventional adult-donated whole blood (AD-WB) in a resource-limited setting, and so concomitant adult whole blood controls were used for comparison. The primary RBC storage lesions investigated were hemolysis and intracellular potassium loss as indicated by supernatant potassium levels.

## MATERIALS AND METHODS

### Sample size and unit selection

The study took place from February to April in 2007 in Mombasa, Kenya. Three units of AD-WB from each of one static and three mobile donation sessions were provided by the Regional Blood Transfusion Centre (RBTC; total of 12 units). No instruction was given about which units from a particular session were to be entered into the study, although it was known that the study was looking at quality and storage issues. Twenty-four consecutive umbilical cord whole blood (UC-WB) donations with a minimum volume of 40 mL were used in the study.

### Collection technique and storage conditions

The RBTC staff collected AD-WB units according to their standard operating procedures: blood donations were collected into single bags designed for a standard 450 mL (±10%) collection and prefilled with 63 mL of CPDA-1 (Medibag; Eastern Medikit Ltd, India); predonation screening of donor hemoglobin (Hb) was performed using the copper sulfate method (specific gravity 1.053, corresponding to a Hb of 12.5 g/dL), and the volume of blood collected was monitored at the time of collection using a spring balance.

UC-WB was collected after delivery of the placenta from consenting mothers delivering at term on the labor ward of Coast Provincial General Hospital, Mombasa. The placenta was placed fetal side down on the absorbent surface of an incontinence pad, which was clipped over a metal hoop. The hoop was attached to a metal rod like a retort stand and the clamped umbilical cord passed through a hole cut into the middle of the incontinence pad. The hoop was raised up (and with it the placenta) and clamped higher up the stand such that the cord hung down with its full length suspended and the umbilical cord vein filled with blood by gravity.

The entire cord was disinfected twice with 70% isopropyl alcohol and then the intended venipuncture site, which is at the distal end of the cord just above where it is clamped, swabbed with 2% povidone iodine tincture. Cord blood was collected by a single venipuncture of the umbilical vein with the 16-gauge needle of a 250-mL single blood collection bag containing 21 mL of CPDA-1 (Macopharma, Twickenham, UK) and drainage of the cord blood into the bag by gravity. Universal precautions and attention to asepsis were observed throughout the procedure.

Screening of all units for human immunodeficiency virus, hepatitis B and C, and syphilis was performed by the RBTC laboratory using their standard methods. In the case of cord blood, a maternal sample taken around the time of delivery was tested. AD-WB and UC-WB units were stored in the same refrigerator at a temperature of 2 to 6°C for 35 days. In the event of main power supply failure, the refrigerator was connected to the hospital diesel generator. The refrigerator temperature was monitored manually twice a day.

### Sampling of blood packs

All blood packs were sampled on Days 2, 7, 14, 21, 28, and 35 after collection (day of collection is Day 1). They were removed from the refrigerator and rocked gently by hand in a plastic tray for 10 minutes to resuspend the cellular components. To reduce the risk of bacterial contamination, which can cause hemolysis, blood packs were sampled on Days 2, 7, 14, and 21 in the following manner: the blood in the pilot tube was stripped back into the main pack three times using a tube stripper, the tubing was clipped twice 15 cm from the distal end of the tube and then cut between the two clips, the two cut ends were rinsed with 70% methanol and the blood pack was placed back in the refrigerator, and the 1 to 2 mL of blood in the removed tube segment was then transferred into a plain test tube.

On Day 28, when pilot tube lengths were too short for the sampling method described above, a sterile spiked sampling coupler (Baxter, Newbury, UK) was inserted aseptically in a laminar flow hood into one of the ports on each pack, and a sample was withdrawn through this. The coupler was left in place and the Day 35 samples were obtained in the same manner.

### Laboratory assays

Unit weights were established by weighing on a tared scale before sampling on Day 2. Hemograms were performed on all samples with an automated cell counter (Nihon Kohden Corp., Shinjuku-ku, Japan). Samples were centrifuged at 3000 × *g* for 5 minutes (Heraeus Instruments, Hanau, Germany) and the supernatant was transferred with a pipette to a different tube, which was centrifuged at 3000 × *g* for an additional 5 minutes. Supernatant (plasma) Hb was assayed with a plasma/low Hb photometer (Hemocue, Angelholm, Sweden) and then the remainder of the supernatant was stored at −20°C. The supernatant (plasma) potassium concentration was assayed at a later date on the thawed plasma samples (Instrumentation Laboratory, Lexington, MA).

### Calculated values and statistical analysis

Donation volumes were estimated by subtracting the CPDA-1 volume from the unit weight and multiplying by 1.06. 12 Supernatant Hb (g/dL), total Hb (g/dL), and hematocrit (Hct) were used to calculate percentage hemolysis according to the formula


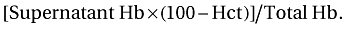


To adjust for the dilution effect of excess CPDA-1 in smaller donations, supernatant potassium concentrations (supernatant K, mmol/L) were standardized by donation volume (L) by estimating the total potassium and dividing by donation volume as follows:


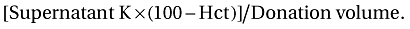


All data were entered into an electronic database and analyzed using statistical software (Stata v9.2, StataCorp, College Station, TX). AD-WB variables were assumed to have a normal distribution and summarized by the mean and standard deviation (SD). The 24 UC-WB donations were ranked according to donation volume and allocated to six equal groups (Table 2). Nonparametric statistics were used to summarize these data and to test for the significance of observed differences between cord blood groups (Kruskal-Wallis) and between adult-donated and cord blood (Wilcoxon rank sum). Binary data were expressed as proportions and observed differences compared for statistical significance with the chi-squared test of association.

**TABLE 2:** **Baseline characteristics of 24 UC-WB donations with stratification by donation volume (see text)**

Group allocation	Unit volume (mL)	Donation volume (mL)	Blood : CPDA ratio	RBC (×10^6^/µL)	Hct (%)	Hb concentration (g/dL)	Total Hb (g)
1	63	42	2.0	2.96	27.0	9.5	5.9
	64	43	2.1	2.78	27.0	9.5	6.1
	64	43	2.1	2.45	25.8	8.8	5.7
	76	55	2.6	3.03	26.9	8.9	6.7
2	79	58	2.8	3.27	32.3	11.4	9.1
	79	58	2.8	3.01	32.2	11.4	9.1
	81	60	2.9	3.88	37.6	12.9	10.5
	81	60	2.9	2.77	27.8	9.9	8.1
3	85	64	3.1	3.4	31.9	11.1	9.5
	91	70	3.3	3.78	35.5	12.1	11.0
	91	70	3.3	4.21	36.8	12.2	11.1
	93	72	3.4	3.38	31.4	10.8	10.0
4	93	72	3.4	3.64	34.7	12.1	11.2
	98	77	3.7	2.78	31.2	10.8	10.6
	98	77	3.7	3.98	37.2	13.1	12.9
	98	77	3.7	3.5	33.9	11.5	11.3
5	100	79	3.8	3.68	38.7	14.0	14.0
	102	81	3.9	2.86	29.9	10.3	10.5
	104	83	4.0	3.36	33.2	11.8	12.3
	106	85	4.0	3.34	34.8	12.2	12.9
6	106	85	4.0	4.4	39.9	13.9	14.7
	123	102	4.9	4.34	33.5	11.5	14.1
	129	108	5.1	4.23	34.9	11.7	15.0
	149	128	6.1	3.28	33.5	11.8	17.6

## RESULTS

Summary baseline (Day 2) characteristics of the 12 AD-WB units are shown in Table 1. Mean donation volume was 387 mL with 8 of the 12 units having a donation volume of less than 405 mL. There was a wide variation in unit Hb concentration (8.6-13.4 g/dL) and total unit Hb content (35-67 g); 2 of the 12 units contained less than 45 g of Hb.

**TABLE 1:** **Summary baseline characteristics of 12 AD-WB units (63 mL of CPDA-1)**

	Unit volume (mL)	Donation volume (mL)	Blood : CPDA ratio (mL)	RBCs (×10^6^/µL)	Hct (%)	Hb concentration (g/dL)	Total Hb (g)
Mean (SD)	450 (29)	387 (29)	6.1 (0.5)	4.07 (0.61)	31.6 (3.5)	11.0 (1.2)	50 (8)
Range	404-498	341-435	5.4-6.9	3.12-5.02	24.5-36.3	8.6-13.4	35-67

Cord blood donation volume ranged from 42 to 128 mL and the ratio of blood to CPDA-1 from 2.0 to 6.1 (Table 2). RBC, Hct, and Hb values for UC-WB all increased with donation volume as the dilution effect of the CPDA-1 was reduced (Table 2), as did supernatant potassium concentration (Table 3).

**TABLE 3:** **Median hemolysis, supernatant potassium (measured and adjusted) for UC-WB (stratified by donation volume, see Table 2 and text), and AD-WB at different sampling time points**

	Hemolysis (%)						Supernatant potassium concentration (mmol/L)						Supernatant potassium/donation volume (mmol/L)					
Sample day	2	7	14	21	28	35	2	7	14	21	28	35	2	7	14	21	28	35
UC-WB 1	0.04	0.08	0.12	0.18	0.40	0.58	4.20	8.72	12.44	15.68	18.38	21.57	4.59	9.25	13.31	16.30	19.42	22.70
UC-WB 2	0.00	0.03	0.14	0.16	0.24	0.56	5.06	10.59	17.39	18.20	23.83	26.95	4.51	9.69	15.23	16.41	21.40	24.16
UC-WB 3	0.05	0.20	0.24	0.31	0.48	0.91	6.01	12.16	17.23	21.61	24.68	29.50	5.19	10.60	14.86	18.87	21.42	26.04
UC-WB 4	0.03	0.15	0.32	0.36	0.51	0.55	5.80	12.09	18.33	22.35	26.27	30.26	4.95	9.96	15.57	18.50	21.36	24.67
UC-WB 5	0.03	0.10	0.25	0.33	0.49	0.62	5.52	12.40	19.32	24.59	27.47	28.86	4.45	9.98	15.30	18.98	21.44	23.50
UC-WB 6	0.06	0.17	0.26	0.27	0.31	0.49	6.73	14.05	20.25	24.20	29.16	32.92	5.14	11.19	15.82	17.76	21.86	25.28
UC-WB all	0.03	0.13	0.22	0.29	0.39	0.57	5.69	11.64	18.05	21.62	25.40	28.86	4.73	10.04	15.10	18.13	21.22	24.47
AD-WB	0.09	0.18	0.28	0.28	0.56	0.61	4.25*	11.37	16.91	22.15	26.19	29.69	3.41*	9.08*	13.43*	17.31	20.42	22.67

* p < 0.05 compared with UC-WB all.

Hemolysis increased throughout the storage period in both AD-WB and UC-WB, reaching median values on Day 35 of 0.61 and 0.57%, respectively (Table 3). There was no significant difference in the median hemolysis rates of cord blood when analyzed by volume or between AD-WB and UC-WB, at any of the sampling points. On Day 28 hemolysis of greater than 0.8% was evident in 2 of the 24 UC-WB units (8%) and 2 of the 12 AD-WB units (17%). An additional 4 UC-WB units showed hemolysis of greater than 0.8% on Day 35. The proportion of units in each group with hemolysis exceeding 0.8% was not significant on either Day 28 or Day 35, but the study had negligible power to detect such differences.

When total supernatant potassium was standardized by donation volume, there was no significant difference between cord blood groups. However, standardized potassium was consistently higher in UC-WB than AD-WB and this was to a significant degree on Days 2, 7, and 14.

## DISCUSSION

This study assessed the storage characteristics of a wide range of volumes of UC-WB collected into a fixed volume of CPDA-1 (21 mL) and refrigerated for 35 days in a resource-limited setting. The findings suggest RBC survival and function, as demonstrated by hemolysis and intracellular potassium loss, are comparable with AD-WB stored under the same conditions and that UC-WB can be stored without difficult and expensive prestorage processing. Cord blood collected and stored in this manner may not be transfused to small children as whole blood if there is a risk of citrate toxicity from a large volume of CPDA-1. However, simpler options for RBC concentration and volume reduction using gravity separation are possible immediately before transfusion when the integrity of a single collection bag can be breached with less risk of bacterial proliferation.^13^,^14^

The increases in hemolysis and supernatant potassium concentration with duration of storage shown here for UC-WB are consistent with previous data,^9^,^15^ our own AD-WB controls, and international norms for whole blood stored in CPDA-1 for 35 days.^12^ Significantly, however, we also show no significant difference in hemolysis rates or adjusted supernatant potassium concentrations for UC-WB over a wide range of cord blood volumes. Previous studies of cord blood collected into fixed volumes of AP solution have not been stratified and analyzed by donation volume in this way.

Our data also demonstrated no significant difference in the hemolysis rates of UC-WB RBCs compared with AD-WB controls at any time point. This is in contrast to cord blood stored as RBCs in extended storage medium, which show significantly higher hemolysis rates at the end of storage (35 days) compared with conventional RBCs.^10^

The higher adjusted supernatant potassium concentrations seen in UC-WB suggest greater intracellular potassium loss from UC-WB RBCs and may indicate a difference in the function of the sodium/potassium adenosine triphosphatase pump in UC-WB RBCs during storage.^12^ However, the difference in supernatant potassium concentrations is not significant after Day 14, the Day 35 levels are comparable to international norms for AD-WB, and intracellular potassium levels are expected to replenish after transfusion.

This study did not investigate other markers of RBC storage such as RBC ATP, reduced glutathione and 2,3-diphosphoglycerate, and osmotic fragility. Previous studies suggest that given the degree of RBC viability demonstrated here, these variables are likely to be within an acceptable range and/or reversible after transfusion.^9^,^15^,^16^ Further work, however, needs to be done and ultimately cord blood RBC survival should be assessed in vivo with clinical trials comparing clinical and hematologic variables before and after cord blood transfusion. Clinical studies in children involving radiolabeled cord blood RBCs would present ethical and practical difficulties in sub-Saharan Africa.

It is noteworthy that after 35 days of storage a proportion of both UC-WB and AD-WB units showed levels of hemolysis higher than international quality standard of 0.8%.^17^ In addition, two-thirds of the AD-WB units provided to the study by the RBTC were below the recommended lower volume limit of 405 mL and were not labeled as such.^17^ This, combined with the likelihood that some donor Hb concentrations were probably less than 12.5 g/dL, resulted in an almost twofold range in total unit Hb and 2 units having a total Hb of less than the international standard of 45 g.^17^ This has implications for pediatric clinical practice, which assumes minimum Hb content for transfused blood and is therefore prescribed using a volume multiplier (20 mL/kg^18^). The AD-WB unit with the lowest Hb content in this study would provide 30% less oxygen-carrying capacity than the average (1.6 g/kg vs. 2.2 g/kg). This may result in poor clinical outcome and/or exposure to further transfusions and raises issues about quality checks, training, and supervision with regard to donor Hb screening and blood collection within the Kenya Blood Transfusion Service.

In conclusion, the findings of this study support the collection of variable volumes of umbilical cord blood into a fixed volume of CPDA-1 and its subsequent refrigerated storage for up to 35 days. This provides further evidence of the feasibility and safety of cord blood RBC transfusion in children with severe anemia in resource-restricted countries. Further work on microbiologic and clinical safety is either planned or under way.

## ABBREVIATIONS:

AD-WB: adult-donated whole blood
AP: anticoagulant-preservative
RBTC: Regional Blood Transfusion Centre
UC-WB: umbilical cord whole blood.
